# Sequential Insertion Heuristic with Adaptive Bee Colony Optimisation Algorithm for Vehicle Routing Problem with Time Windows

**DOI:** 10.1371/journal.pone.0130224

**Published:** 2015-07-01

**Authors:** Sana Jawarneh, Salwani Abdullah

**Affiliations:** Data Mining and Optimisation Research Group, Centre for Artificial Intelligence Technology, Universiti Kebangsaan Malaysia, 43600 Bangi, Selangor, Malaysia; Beihang University, CHINA

## Abstract

This paper presents a bee colony optimisation (BCO) algorithm to tackle the vehicle routing problem with time window (VRPTW). The VRPTW involves recovering an ideal set of routes for a fleet of vehicles serving a defined number of customers. The BCO algorithm is a population-based algorithm that mimics the social communication patterns of honeybees in solving problems. The performance of the BCO algorithm is dependent on its parameters, so the online (self-adaptive) parameter tuning strategy is used to improve its effectiveness and robustness. Compared with the basic BCO, the adaptive BCO performs better. Diversification is crucial to the performance of the population-based algorithm, but the initial population in the BCO algorithm is generated using a greedy heuristic, which has insufficient diversification. Therefore the ways in which the sequential insertion heuristic (SIH) for the initial population drives the population toward improved solutions are examined. Experimental comparisons indicate that the proposed adaptive BCO-SIH algorithm works well across all instances and is able to obtain 11 best results in comparison with the best-known results in the literature when tested on Solomon’s 56 VRPTW 100 customer instances. Also, a statistical test shows that there is a significant difference between the results.

## Introduction

The vehicle routing problem (VRP) is the generic name given to all types of problems that involve a set of routes for a fleet of vehicles that use one or more depots to serve a geographically delimited town or set of customers. The VRP was proposed by Dantzig and Ramser in 1959 [1]. Its main objective is to serve all customers while imposing a limited demand on the minimum number of vehicles and/or total cost.

One of the extensions of the VRP is the vehicle routing problem with time window (VRPTW), which is concerned with a predefined time interval called the time window. In this scenario, a vehicle can visit a location only in a specified time window. In other words, after the latest time set for the time window, a vehicle is no longer accommodated. Each vehicle starts and ends at a central depot and has a limited capacity. However, if a vehicle arrives at a destination before the earliest stated time, then it must wait until the beginning of the time window.

The main objective of the VRPTW is to service all the requirements of the customers while minimising the total distance travelled by all the vehicles without violating certain constraints. These constraints include the following: (1) each customer is visited only once, (2) the sum of all the demands on a given vehicle must not be more than the maximum capacity of the vehicle, and (3) the vehicle must arrive after the earliest time and visit all the customers within a specific predefined time.

The VRPTW has been tackled by using exact approaches [2, 3], heuristic approaches [4–8], and metaheuristic approaches [9, 10]. It has also been the subject of population-based approaches, including the particle swarm optimisation (PSO) algorithm [11, 12], artificial bee colony (ABC) algorithm [13], genetic algorithm [14], and ant colony optimisation (ACO) [15]. The present study uses an algorithm based on an adaptive bee colony optimisation (BCO) algorithm. The BCO algorithm was introduced by Teodorovic et al. [16]. Some experiments that tackle Solomon instances of the VRPTW have been presented. A new approach has also been proposed to improve the effectiveness and robustness of the BCO algorithm using the online (self-adaptive) parameter tuning strategy. In that work, two strategies are used for parameter tuning: offline parameter initialisation and an online parameter tuning strategy. The values of different parameters in offline parameter initialisation are fixed before the execution of the metaheuristic, whereas the values of the parameters in the online approach are controlled and updated dynamically or adaptively during the execution of the metaheuristic [17].

A review of the literature shows that the initial population used in most of the cases is generated randomly or through a greedy approach; few researchers focus on generating an initial population, where diversification plays a crucial role in the performance of population-based algorithms [17]. Therefore, we propose a sequential insertion heuristic (SIH) to achieve this diversification. The experimental results show that the online parameter tuning strategy (adaptive BCO algorithm) improves the results of the BCO algorithm. In addition, the adaptive BCO algorithm performs better when SIH is used for its initial population than when the greedy heuristic approach is used. A comparison with other state-of-the-art approaches indicates that our adaptive BCO algorithm obtains the best results in terms of achieving the main objective of the VRPTW, which is to reduce the distance travelled.

The remainder of this paper is organised as follows: Section 2 presents the VRPTW formulations. Section 3 discusses the Solomon benchmark datasets for the VRPTW. Section 4 proposes the BCO algorithm for the VRPTW. Section 5 presents the population initialisation strategy. Section 6 discusses the experimental and comparative results. Section 7 concludes the paper.

## VRPTW Formulations

This section presents a mathematical formulation of the VRPTW. We start with a natural understanding of the problem before arriving at a precise formulation. The VRPTW constraints contain a set of matching vehicles, an essential depot node, a specific number of nodes of scattered customers, and a network connecting the customers to the depot.

**Fig 1:**
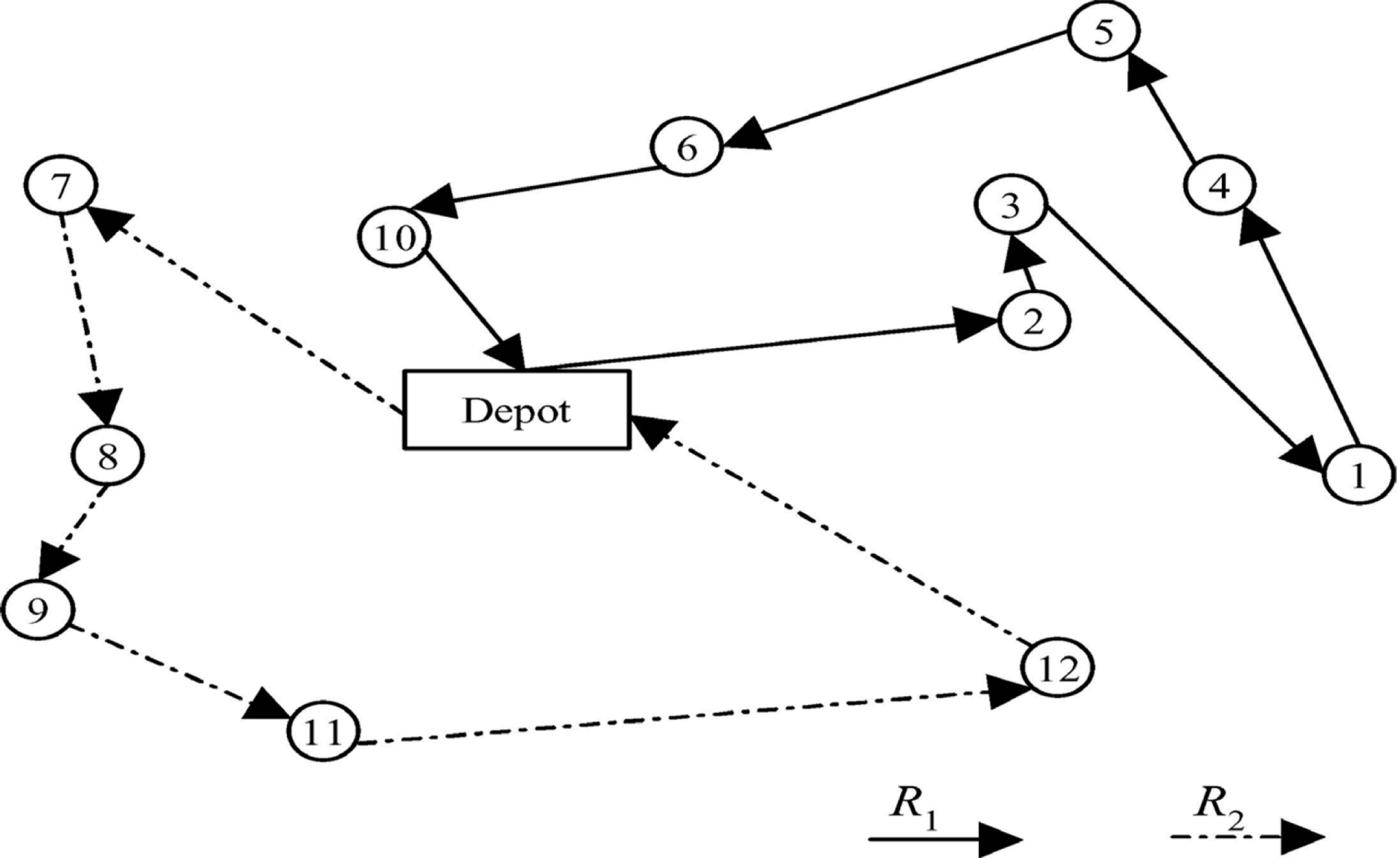
Sample solution of a simple vehicle routing problem.

Fig 1 shows a graphic representation of a simple VRPTW and its solution. R1 and R2 represent the two routes, and every number in the network represents a customer. The arrows connect all the customers, and every vehicle must service them by starting from and finishing at the depot.

Based on Solomon’s 1987 model, our formulation presupposes *N*+1 customer and *K* vehicles. Customer 0 is located at the depot node, and each arc in the network represents a connection between two nodes and specifies the direction of travel. Every route starts from the depot, passes through all the customers, and then returns to the depot. Every vehicle represents one route in the network.

Cost *c*_*ij*_ and travel time *t*_*ij*_ are related to every arc in the network. In Solomon’s 56 VRPTW 100 customer instances, the speed of the vehicle is assumed to be unitary; that is, only one unit of time is spent to travel one unit of distance.

Every vehicle has the same capacity *q*_*k*_, and each customer with demand *m*_*i*_ must be visited only once by one of the vehicles. The summation of the capacity of all the demands on the route must be equal to or less than the *q*_*k*_ of vehicle *k* on the route being travelled and no vehicles should be overloaded.

The time window constraints are indicated by a predefined time interval, an earliest arrival time (*e*_*i*_), and a latest arrival time (*l*_*i*_). The vehicle must reach the customer before the latest arrival time; if it arrives before the earliest arrival time, then it must wait. A service time for each customer for a given loading/unloading time is also considered. Solomon assumes this loading/unloading time to be unique regardless of the quantity of the demands. Vehicles are assumed to finish their route within the total route time, which is essentially the time window. The VRPTW has the following variables and parameters:

- xijk = 01if there is no arc from node i to node jotherwise i≠j, i, j∈{0,1,2,………N}
- *t*_*i*_ *arrival time at point i*
- *w*_*i*_ *waiting time at point i*
- *K number of vehicles*
- *N number of customers (0 denotes the central depot)*
- *c*_*ij*_ *cost incurred in arc from customer i to customer j*
- *t*_*ij*_ *travel time between customer i and customer j*
- *m*_*i*_ *demand at customer i*
- *q*_*k*_ *capacity of vehicle k*
- *e*_*i*_ *earliest arrival time at customer i*
- *l*_*i*_ *latest arrival time at customer i*
- *f*_*i*_ *service time at customer i*
- *r*_*k*_ *maximum route time for vehicle k*

The explanations for the VRPTW formulation are as follows:

$$\text{Minimise}\overset{N}{\underset{i=0}{\sum }}\overset{N}{\underset{j=0,j\neq i}{\sum }}\overset{K}{\underset{k=1}{\sum }}c_{ij}x_{ijk}$$ (1)

Subject the equation to

$$\overset{K}{\underset{k=1}{\sum }}\overset{N}{\underset{j=1}{\sum }}x_{ijk}\leq K\;\;\;\;\;\;for\;i=0$$ (2)

$$\overset{N}{\underset{j=0}{\sum }}x_{ijk}=\overset{N}{\underset{j=1}{\sum }}x_{jik}\;\leq 1\;\;\;\;\;\;for\;i=0\;and\;\;k\;\in \left\{1,2\ldots \ldots .,k\right\}$$ (3)

$$\overset{K}{\underset{k=1}{\sum }}\overset{N}{\underset{j=0,j\neq i}{\sum }}x_{ijk}=1\;\;\;\;\;\;for\;i\;\in \left\{1,2,\ldots \ldots ..,N\right\}$$ (4)

$$\overset{K}{\underset{k=1}{\sum }}\overset{N}{\underset{i=0,j\neq i}{\sum }}x_{ijk}=1\;\;\;\;\;\;for\;j\;\in \left\{1,2,\ldots \ldots ..,N\right\}$$ (5)

$$\overset{N}{\underset{i=1}{\sum }}m_{i}\overset{N}{\underset{j=0,j\neq i}{\sum }}x_{ijk}\leq q_{k}\;\;\;\;\;\;for\;k\in \left\{1,2,\ldots \ldots ..,K\right\}$$ (6)

$$\overset{N}{\underset{i=1}{\sum }}\overset{N}{\underset{j=0,j\neq i}{\sum }}x_{ijk}\left(t_{ij}+f_{i}+\;w_{i}\right)\leq r_{k}\;\;\;\;\;\;for\;k\in \left\{1,2,\ldots \ldots ..,K\right\}$$ (7)

$$t_{0}=w_{0}=f_{0}=0$$ (8)

$$\overset{K}{\underset{k=1}{\sum }}\overset{N}{\underset{i=0,j\neq i}{\sum }}x_{ijk}\left(t_{i}+t_{ij}+f_{i}+\;w_{i}\right)\leq t_{j}\;\;\;\;\;\;for\;j\in \left\{1,2,\ldots \ldots ..,N\right\}$$ (9)

$$e_{i}\leq (t_{i}+\;w_{i})\leq l_{j}\;\;\;\;\;\;for\;i\in \left\{1,2,\ldots \ldots ..,N\right\}$$ (10)

$$c_{ij}=\sqrt{{\left(i_{x}-\;j_{x}\right)}^{2}+{\left(i_{y}-\;j_{y}\right)}^{2}}$$ (11)

Eq 1 is the main objective function of the VRPTW problem. Constraint (2) indicates the maximum K routes or vehicles going out of the depot. Eq 3 specifies that every route must start and finish at the main depot. Eqs 4 and 5 ensure that every customer is visited only once by exactly one vehicle. Eqs 6 and 7 define the capacity and maximum travel time constraints, respectively. Constraints (8), (9), and (10) explain the time window. Eq 11 calculates the cost of travel, where *i*_*x*_ is the coordinate *x* for customer *I* and *i*_*y*_ is the coordinate *y* for customer *i*.

## Solomon Benchmark Datasets for the VRPTW

In 1987, Solomon proposed six benchmark categories for the VRPTW (56 VRPTW 100 customer instances), which have since been widely used for different heuristics [6]. The present study uses these instances to test the performance of the proposed algorithms. The instances differ in the total number of vehicles, travelling time, vehicle capacity, customer location, and suitable time of service. In other words, every customer has their own time window, location (given as x and y coordinates), quantity of demand, ready time, due time, and service time needed.

All instances presuppose 100 customers and a travelling time between customers that is equal to the corresponding Euclidean distance. The 56 instances are divided into six categories based on the pattern of the customer locations and time windows. These categories are named C1, C2, R1, R2, RC1, and RC2. Category C consists of clustered customers, category R consists of remotely located customers, and category RC consists of a mix of remotely located and clustered customers.

The geographical allocation determines the travelling distance between customers. Customers in the cluster category are located closer to one another, and their travelling distance is shorter than in other categories. In contrast, customers in the remote category are located far from one another, such that their traveling distance is longer than in the category C instances. Category C instances are by nature easy to solve because of the short distances between the customers, whereas obtaining a perfect sequence of customers along each route in the case of the R category instances is difficult.

**Fig 2:**
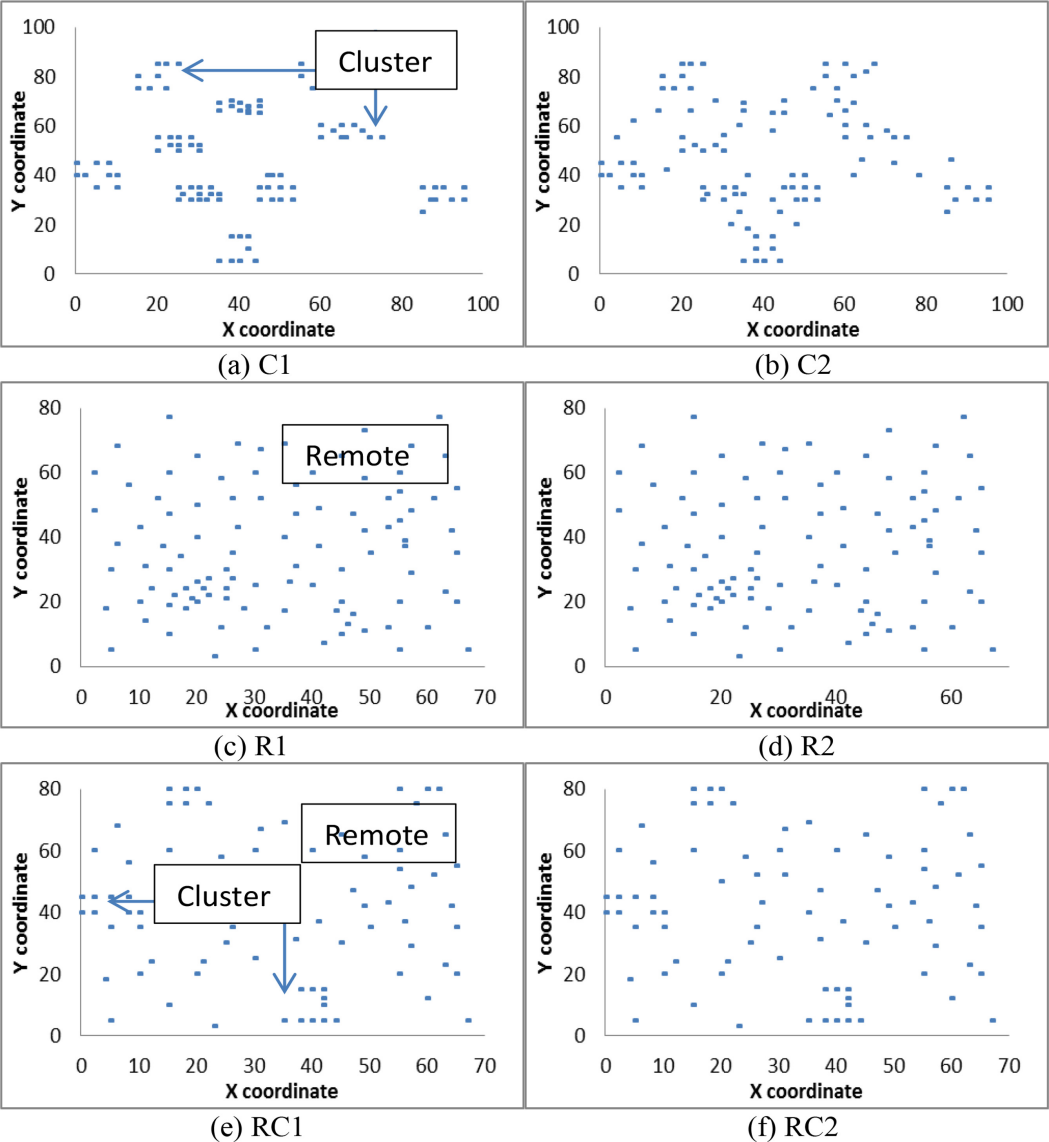
Distribution of customer locations in problem categories C1, C2, R1, R2, RC1, and RC2.

In Fig 2, the x-y coordinate represents the distribution of customer locations in the six different categories, namely, C1, C2, R1, R2, RC1, and RC2. Fig 2a, 2c and 2e are labelled with ‘cluster’ and/or ‘remote’ to identify the distribution of customers. Fig 2e shows that the RC category has both R and C type patterns.

The instances are further categorised according to the time window constraints. The instances in categories C1, R1, and RC1 have a relatively short time window, whereas the instances in categories C2, R2, and RC2 have a relatively long time window. The time windows for instances R1 and RC1 are randomly produced.

## Proposed BCO Algorithm for the VRPTW

This section presents the BCO algorithm, adaptive BCO algorithm, parameter settings, and neighbourhood structures.

### 4.1 BCO Algorithm

The swarm intelligence characteristics inspired a number of researchers to use such behaviour in algorithms for solving optimisations problems, including Ant Colony Optimization (ACO) [18], Particle Swarm Optimization (PSO) [19], Firefly Algorithm (FA) [20] and honeybees algorithms [21].

The BCO algorithm, which was introduced by Teodorovic et al. [16] as a new field of swarm intelligence, simulates the foraging behaviour of honeybees in nature. The algorithm has two main passes: the forward pass and backward pass. In the forward pass (denoted by the neighbourhood structure in our method), all the bees fly to the field and explore the search space by exchanging information about food sources, after which they return to the hive with the collected amount of nectar (denoted by distance in our method). In the backward pass, each bee chooses to continue its own exploration either as a recruiter or as a follower based on the information collected during the forward pass. When a bee becomes a recruiter, it continues its search based on the available information; conversely, when a bee becomes a follower, it selects one recruiter to guide its search process (by a roulette wheel selection) [22–25].

**Fig 3:**
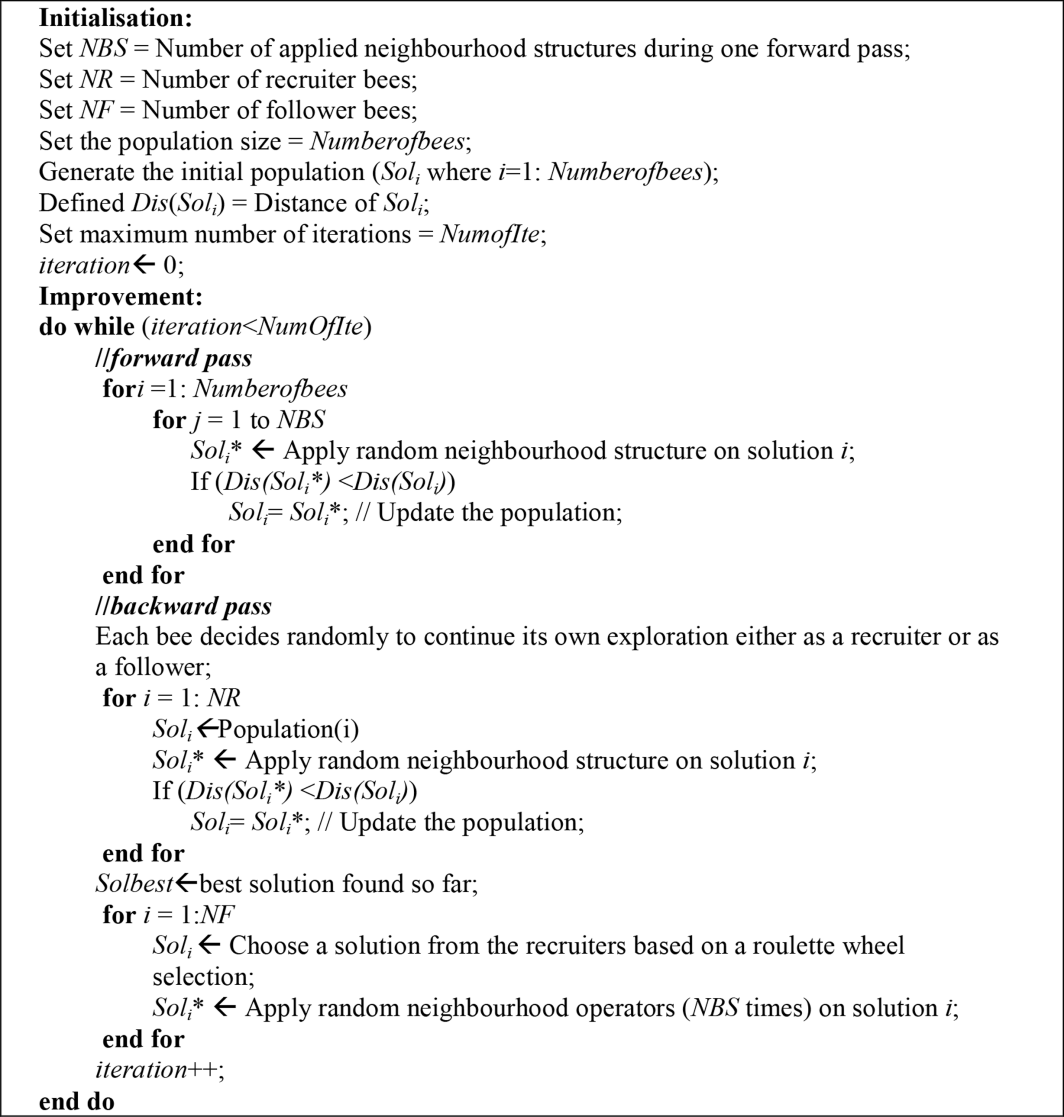
Pseudo-code for BCO algorithm.

Fig 3 shows the pseudo-code for the BCO algorithm, which starts with the initialisation of the parameters, the values of which must be set before the algorithm is executed (offline strategy). A random solution is created for each bee in the hive (the number of bees is equal to the size of the population) as follows: All the routes are taken as empty, and the number of routes is equal to the maximum number of vehicles. A random route is chosen for all the customers, one that satisfies all the hard constraints. If the placement of all the customers is feasible on the routes, then the solution is returned; if not, then customers are randomly added or removed from the routes until a feasible solution is found.

In the *forward pass*, all bees in the hive start their own search without any information by applying a number of random neighbourhood structures (*NBS*). Then, each bee keeps the best solution it finds during the search.

In the *backward pass*, all bees return to the hive, and then each bee decides to continue its own exploration either as a recruiter or as a follower based on the information it collected from the *forward pass* stage. This decision is taken with the probability shown in Eq 2, in which exploration as a recruiter leads to a higher probability of better solutions being found.

$$\begin{array}{cc} p_{i}= & \frac{fi}{\sum_{i=1}^{SN}fi} \end{array}$$ (12)

where SN = population size and *fi* is a fitness function of the *i* ^*th*^ solution.

### 4.2 Adaptive BCO Algorithm

**Fig 4:**
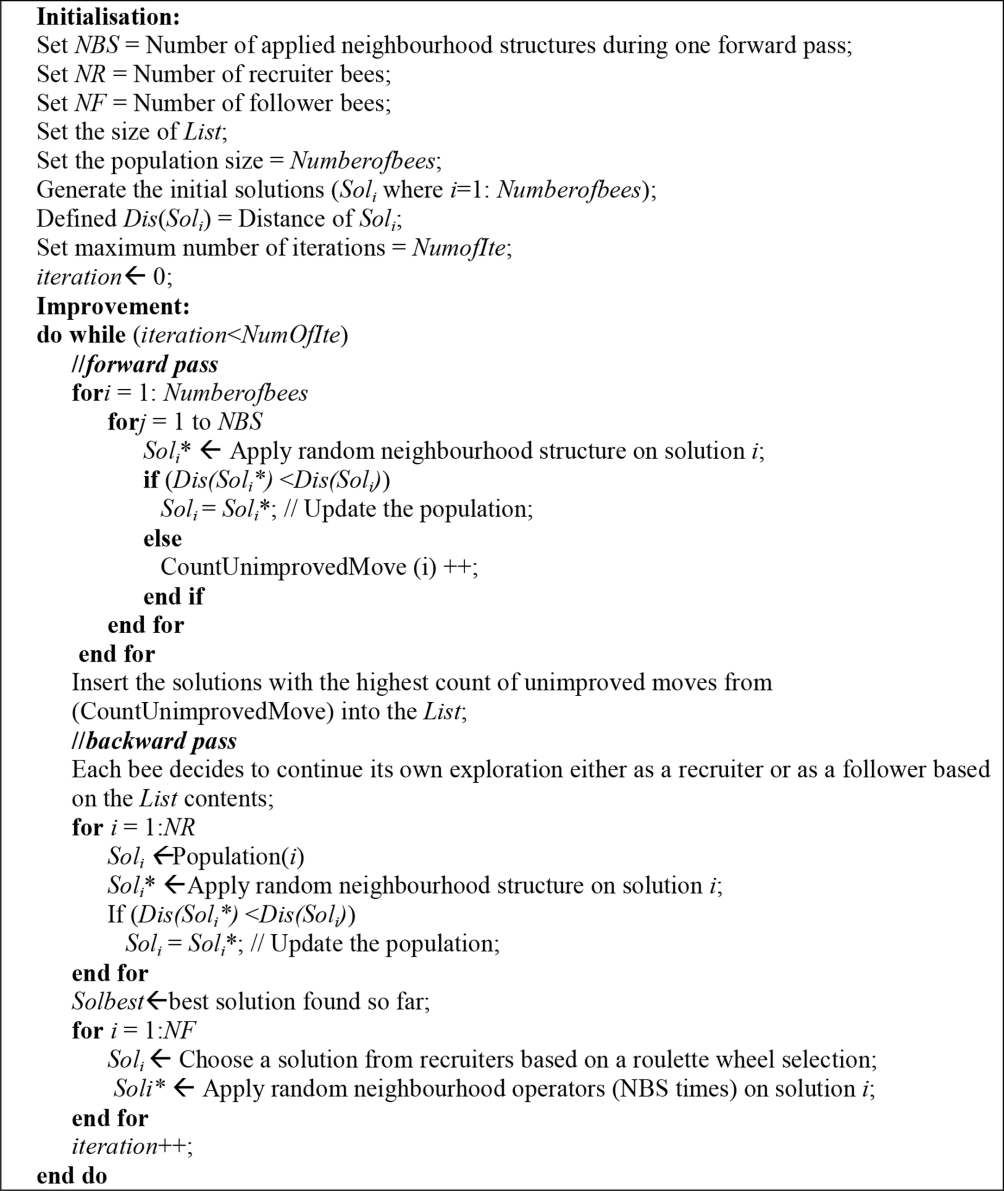
Pseudo-code for adaptive BCO algorithm.

In the adaptive BCO algorithm, each bee in the *backward pass* chooses to continue its own exploration either as a recruiter or as a follower depending on the value of an unimproved counter (Fig 4). The counter increases when the solution does not improve even after a number of neighbourhoods are applied on the forward pass. Solutions with the highest value of unimproved moves are inserted in a previously defined list. The size of the list is chosen based on the primary experiment that defines 10 as the maximum number of follower bees, which in turn is based on the list (List). Solutions in the list are considered as the follower bees and the rest are the recruiters.

The BCO algorithm uses the offline parameter setting, whereas the adaptive BCO algorithm uses the online parameter tuning strategy and the unimproved counter parameter (CountUnimprovedMove), as shown in Fig 4. The performance of the algorithm depends on the number of follower bees (NF) and is very difficult to tune because of the different datasets needed to generate different settings. Here, we propose using the online parameter tuning strategy, in which the number of follower bees is defined based on an adaptive list (List) that saves the unimproved solutions of the population.

## Generating the Initial Population

### 5.1 Greedy Heuristic (GH)

Population-based algorithms usually use a greedy approach to initialise the population of solutions and to improve the solutions by applying an algorithm with neighbourhood exchanges or local searches. The greedy approach used to initialise the population works as follows:

For every solution in the population, the following must be conducted:

1. All the routes are taken as empty; the number of routes is equal to the maximum number of vehicles.
2. A random route that satisfies all the hard constraints must be chosen for all the customers. If all the customers are feasibly located on the routes, then the solution is saved; otherwise, the unallocated customers are inserted into a new route.

### 5.2 Sequential Insertion Heuristic (SIH)

Solomon [6] divides the VRP tour-building algorithms into either parallel or sequential methods. Parallel procedures are built through the concurrent construction of routes, where the number of routes is limited to a predetermined number or formed freely. Sequential procedures construct one route at a time until all the customers are scheduled. Solomon [6] derives the algorithm from five initial evaluated solution heuristics. The SIH is very effective in terms of the quality of the solution and the computational time required to find the solution [26].

The initialisation criteria for finding the initial solutions refer to the process of choosing the first customer to be inserted in the route. The most commonly used initialisation criterion is either the farthest unrouted customer, the customer with the earliest deadline, or the earliest and latest acceptable arrival time. The first customer inserted in a route is called the ‘seed customer’. When the seed customer is chosen and inserted in a route, the SIH algorithm considers the insertion place for the unrouted nodes that minimises the additional distance and time necessary to include a customer in the recent and partially created route, which, in turn, determines the insertion criteria. The selection criteria in the next step attempt to maximise the advantage resulting from inserting a customer in the current partial route rather than in a new direct route.

## Experimental Results

The results for each benchmark instance are represented by the distance, number of vehicles, and average distance of 31 runs, with a focus on minimising the distance. The proposed algorithm is implemented using JAVA programming language and performed on an Intel Core i3 processor. The execution times are between 30 seconds and 15 minutes.

### 6.1 Parameter Settings

Preliminary experiments are conducted to obtain the most appropriate values for the number of iterations, the number of recruiter (NR) and follower (NF) bees, and the final settings of the parameters (Table 1).

**Table 1:** Parameter settings for BCO and adaptive BCO algorithms.

	Parameters			
Algorithms	Number of bees	NR	NF	Number of iterations
**BCO**	50	45	Numberofbees-NR	500
**Adaptive BCO**	50	Numberofbees-NF	Size of the List	500

### 6.2 Neighbourhood Structures

To describe the neighbourhood structures used in this study, the following are assumed:

- *w*, *x*, *y*, and *z* are customers in the same route visited in the sequence {*w*→*x*→*y*→*z*}.
- *s*, *t*, *u*, and *v* are customers in another route visited in the sequence {*s*→*t*→*u*→*v*}.

The following neighbourhood structures are used to generate a neighbour solution:

- *NBS1*: One shift in the same route →*x* is removed from the path and inserted after *z*; the new sequence is {*w*→*z*→*y*→*x*}.
- *NBS2*: Two shifts in the same route →*w* and *x* are removed from the path and inserted after y and z; the new sequence is {*y*→*z*→*w*→*x*}.
- *NBS3*: One shift in a different route →*w* is removed from the first path and inserted in the other path; the new sequence of the second path is {*w*→*s*→*t*→*u*→*v*}.
- *NBS4*: Two shifts in different routes →*w* and *x* are removed from the first path and inserted in the other path; the new sequence of the second path is {*w*→*x*→*s*→*t*→*u*→*v*}.
- *NBS5*: One swap in the same route →*w* and *y* are exchanged; the new sequence is {y→z→*w*→*x*}.
- *NBS6*: Two swaps in the same route →*w* and *x* are exchanged with *y* and *z*; the new sequence is {*w*→*x*→*y*→*z*}.
- *NBS7*: One swap in a different route →*x* from the first path is exchanged with *t* from the second; the new sequences are {*w*→*t*→*y*→*z*} and {*s*→*x*→*u*→*v*}.
- *NBS8*: Two swaps in different routes →*x* and *y* from the first path are exchanged with *t* and *u* from the second; the new sequences are {*w*→*t*→*u*→*z*} and {*s*→*x*→*y*→*v*}.

### 6.3 Comparison of BCO and Adaptive BCO

The results show that the adaptive BCO algorithm performs better than the BCO algorithm in terms of distance, number of vehicles, and average distance of 31 runs (Table 2). The performance of the adaptive BCO algorithm is attributed to its use of the online (self-adaptive) parameter tuning strategy, which helps to improve the efficiency and robustness of the algorithm. The adaptive BCO algorithm clearly produces good results under different hard constraints in different instances.

**Table 2:** Comparison of BCO and adaptive BCO algorithms for VRPTW.

		BCO			Adaptive BCO			
			Distance			Distance		
No	Instance	N.V	Best	Avg	N.V	Best	Avg	p-value
0	R1-01	20	1648.08	1661.49	20	1644.50	1654.43	0.0062
1	R1-02	18	1483.43	1506.17	18	1481.46	1492.45	4.3685E-7
2	R1-03	16	1274.47	1294.17	14	1252.07	1264.44	2.2756E-13
3	R1-04	11	1057.9	1105.03	11	1035.41	1066.04	1.8481E-13
4	R1-05	16	1382.4	1418.07	15	1367.3	1391.06	9.9727E-12
5	R1-06	14	1285.69	1319.28	13	1262.78	1290.22	1.5675E-10
6	R1-07	12	1133.42	1171.56	11	1122.15	1144.76	3.4259E-9
7	R1-08	11	1028.2	1060.13	10	1013.6	1030.05	1.2694E-9
8	R1-09	14	1210.52	1245.87	13	1181.01	1213.14	9.3850E-10
9	R1-10	13	1161.76	1188.78	12	1117.01	1148.63	3.6577E-13
10	R1-11	13	1137.98	1175.30	12	1099.26	1133.51	3.1711E-14
11	R1-12	11	1032.07	1076.22	11	1009.68	1039.33	3.6167E-12
12	R2-01	8	1211.71	1241.25	7	1197.87	1212.23	6.1811E-9
13	R2-02	8	1112.5	1156.38	6	1107.51	1123.12	2.6468E-10
14	R2-03	7	994.77	1024.89	4	984.85	999.54	9.5324E-10
15	R2-04	6	917.67	951.46	4	803.47	840.90	2.1009E-32
16	R2-05	7	1017.7	1064.54	6	993.42	1012.96	7.8857E-18
17	R2-06	6	985.64	1018.67	4	959.98	982.33	3.6997E-12
18	R2-07	4	974.07	986.65	3	934.37	943.76	1.2932E-19
19	R2-08	5	882.58	922.9	3	747.15	822.57	1.7420E-19
20	R2-09	6	981.54	999.35	5	944.48	960.25	1.2680E-19
21	R2-10	7	1007.95	1047.52	5	994.29	1017.15	2.4495E-9
22	R2-11	5	882.86	961.65	4	837.72	873.27	5.8685E-20
23	C1-01	10	828.94	903.63	10	828.94	828.94	1.6839E-15
24	C1-02	11	917.39	958.27	10	828.94	845.55	2.7939E-32
25	C1-03	10	939.03	967.26	10	849.86	876.02	9.7364E-27
26	C1-04	10	946.75	973.71	10	886.24	922.50	4.9519E-19
27	C1-05	11	868.14	915.03	10	828.94	831.13	6.7861E-30
28	C1-06	10	830.46	910.91	10	828.94	833.14	2.9371E-18
29	C1-07	11	862.82	923.86	10	828.94	830.09	7.1707E-30
30	C1-08	10	866.98	936.32	10	833.98	853.39	7.0385E-22
31	C1-09	10	905.02	950.57	10	852.98	883.69	7.7770E-23
32	C2-01	3	591.56	591.56	3	591.56	591.56	0.0176
33	C2-02	3	591.56	623.18	3	591.56	593.16	3.7707E-18
34	C2-03	3	602.17	646.01	3	600.54	616.39	1.0483E-6
35	C2-04	4	763.18	838.80	3	610.01	648.57	3.6223E-32
36	C2-05	3	589.72	606.48	3	588.88	596.10	0.0004
37	C2-06	3	600.00	621.78	3	588.88	601.49	1.2490E-7
38	C2-07	3	593.66	607.82	3	589.58	601.60	0.0134
39	C2-08	3	599.76	628.01	3	591.65	613.47	0.0008
40	RC1-01	16	1667.5	1699.42	15	1644.82	1674.45	1.1576E-5
41	RC1-02	15	1514.38	1549.11	14	1495.18	1508.13	4.3804E-13
42	RC1-03	12	1364.45	1401.33	12	1307.59	1356.70	8.6407E-10
43	RC1-04	11	1242.92	1272.06	10	1215.82	1233.24	1.7411E-11
44	RC1-05	17	1594.46	1625.38	15	1539.48	1587.19	2.2113E-9
45	RC1-06	13	1443.34	1488.57	13	1402.4	1444.13	5.0089E-12
46	RC1-07	12	1306.31	1370.86	12	1258.31	1309.30	3.1113E-14
47	RC1-08	12	1226.78	1274.43	11	1171.61	1222.96	5.0456E-11
48	RC2-01	9	1338.75	1370.63	6	1321.07	1340.22	4.1694E-11
49	RC2-02	8	1189.25	1231.83	5	1185.44	1190.93	2.9019E-12
50	RC2-03	6	1036.92	1070.48	4	1030.94	1030.05	1.6385E-10
51	RC2-04	4	932.77	967.00	4	864.6	918.12	1.7818E-17
52	RC2-05	8	1244.11	1274.25	7	1211.01	1254.57	0.0004
53	RC2-06	6	1140.23	1192.54	6	1112.38	1153.15	3.9012E-10
54	RC2-07	6	1085.86	1122.91	5	1042.65	1063.83	1.0136E-13
55	RC2-08	7	954.94	976.10	4	878.87	913.69	3.0185E-16

To show the significant difference between the two algorithms, we perform the t-test and p-value for the Solomon instances, as shown in the last column of Table 2. The results for the adaptive BCO algorithm indicate significant differences in all instances (100%), which are below the significant level (α = 0.05) in terms of distance. Furthermore, the number of vehicles is less than or equal after the modification.

**Fig 5:**
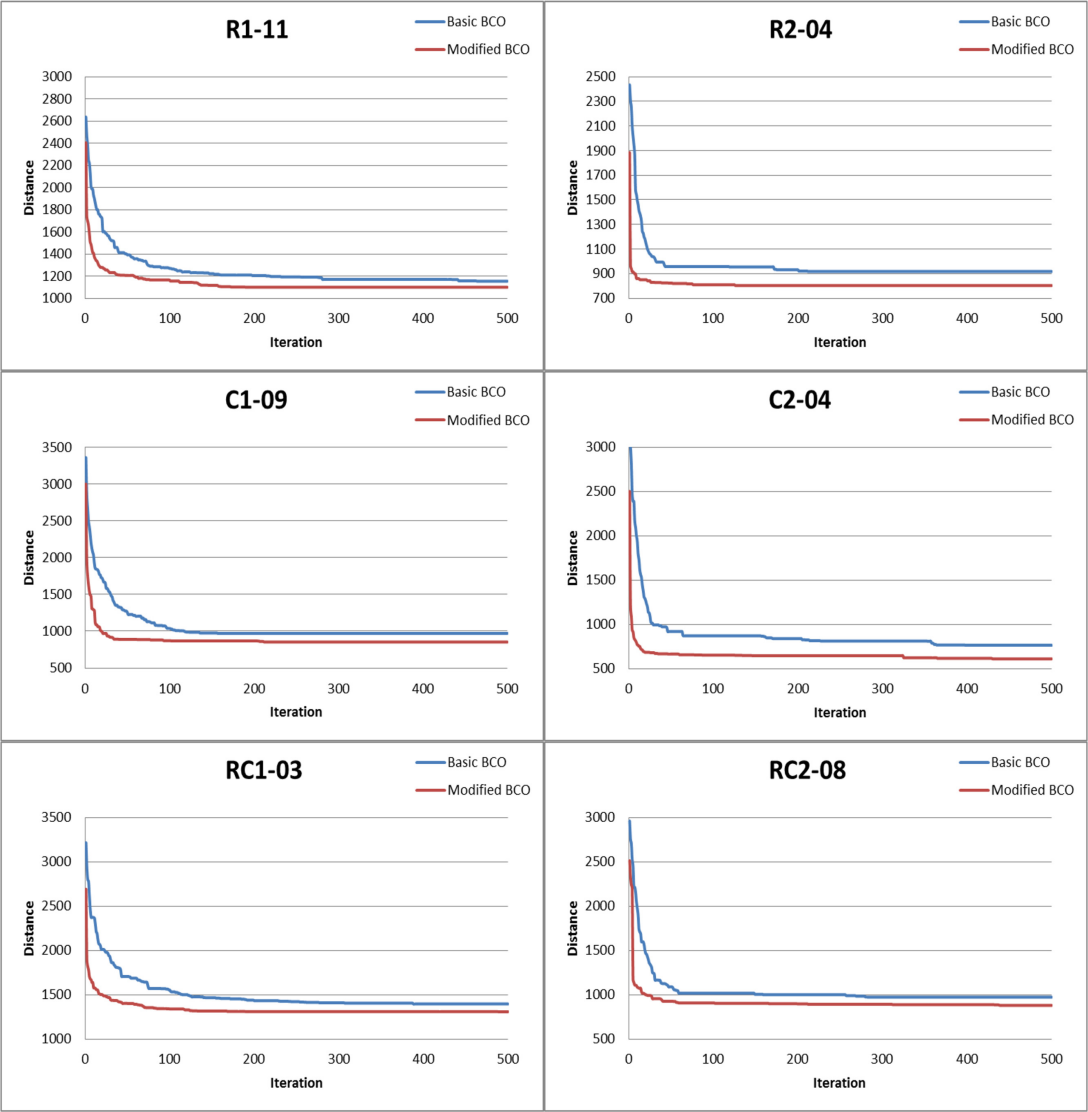
Comparison of solution performance for distance between BCO and adaptive BCO algorithms.

Fig 5 illustrates the convergence curves of the best runs of the BCO and adaptive BCO algorithms for six randomly selected instances: R1-11, R2-04, C1-09, C2-04, RC1-03, and RC2-08 (i.e., one from each category). The distance oscillates to some extent, but decreases as the number of iterations increases in both algorithms. We also observe that the results of the adaptive BCO algorithm can indicate lower distances for all instances compared with the results of the BCO algorithm.

**Fig 6:**
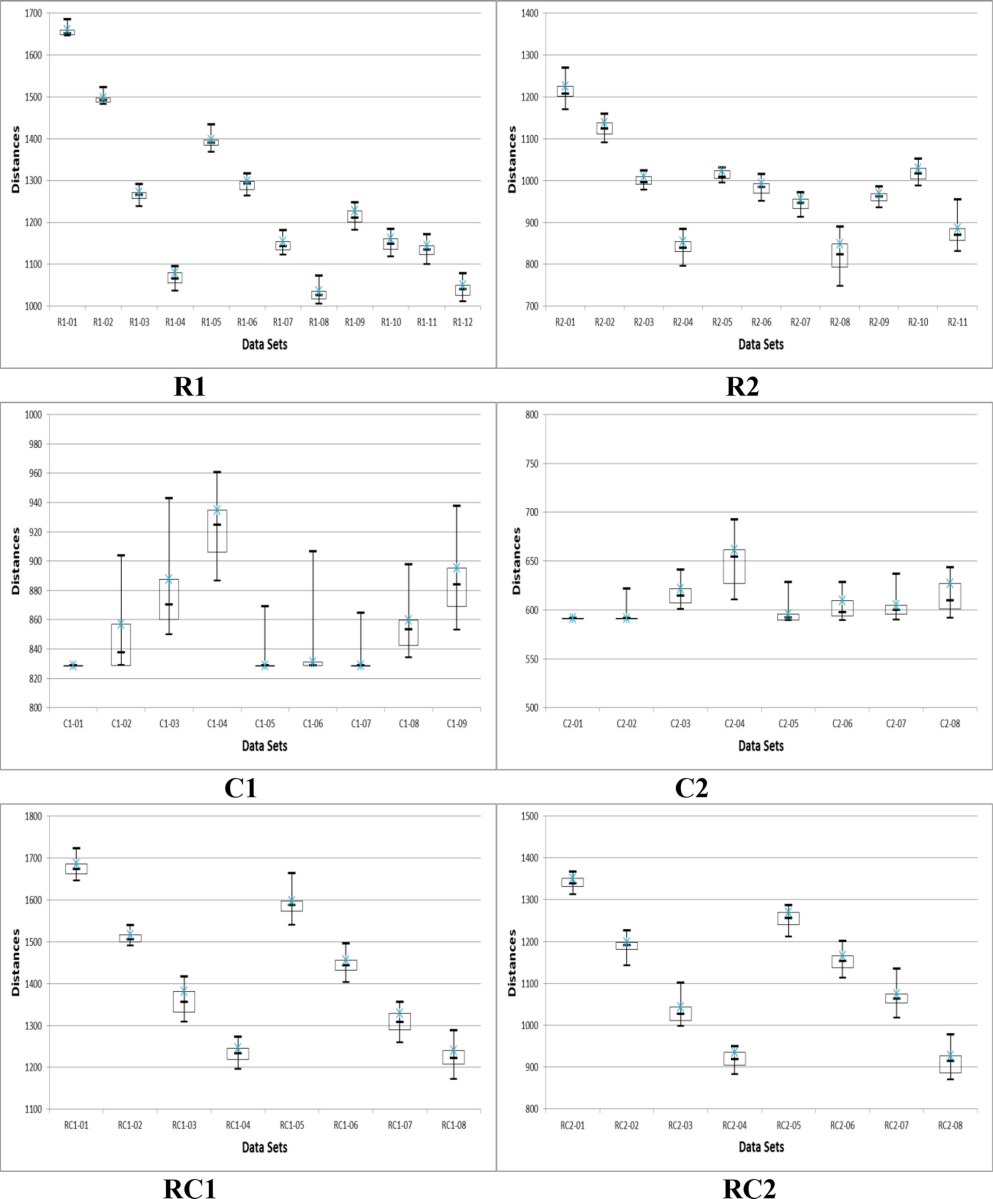
Box and Whisker plots for Solomon datasets for adaptive BCO algorithm.

To study the consistency and reliability of the results achieved by the adaptive BCO algorithm and to obtain a sense of the solution distribution, we examine the maximum, median, minimum, lower, and upper using box and whisker plots for Solomon’s 56 datasets (Fig 6). Each box plot in Fig 6 represents the distribution of the results of 31 runs. The horizontal line inside the box represents the median, and the upper and lower ends of the box represent the upper and lower quartiles. The line appendages represent the spread, shape of distribution, and outside values.

Fig 6 shows that the results obtained by the adaptive BCO algorithm for the 31 runs are highly consistent. All the categories of both types 1 (C1, R1, RC1) and 2 (C2, R2, RC2) indicate a small variance in the solutions. The adaptive BCO algorithm generally results in high-quality solutions, where the minimum and median are very close to each other in almost all the tested datasets, proving the robustness of the proposed algorithm. The discussion above shows that the adaptive BCO algorithm is more effective than the BCO algorithm. Therefore we use the former with SIH to attempt to further improve the results, as described in the next subsection.

### 6.4 Comparison of the Adaptive BCO Algorithm with the Greedy Approach Heuristic (Adaptive BCO-GH) and the Adaptive BCO Algorithm with SIH (Adaptive BCO-SIH)

The results show that the adaptive BCO-SIH algorithm performs better than the adaptive BCO-GH algorithm in terms of distance, number of vehicles, and average distance of 31 runs (Table 3). The performance of the adaptive BCO-SIH algorithm is a result of its use of the SIH, which improves the diversification of the initial population of the adaptive BCO algorithm. The adaptive BCO-SIH algorithm clearly produces good results under different hard constraints in different instances.

**Table 3:** Comparison of results of adaptive BCO-GH and adaptive BCO-SIH algorithms for VRPTW.

		Adaptive BCO-GH			Adaptive BCO-SIH		
			Distance			Distance	
No	Instance	N.V	Best	Avg	N.V	Best	Avg
0	R1-01	20	1644.50	1654.43	20	**1643.18**	1652.93
1	R1-02	18	1481.46	1492.45	18	**1476.11**	1490.32
2	R1-03	14	1252.07	1264.44	14	**1245.86**	1259.53
3	R1-04	11	1035.41	1066.04	11	**1026.91**	1053.87
4	R1-05	15	1367.3	1391.06	15	**1361.39**	1386.75
5	R1-06	13	**1262.78**	1290.22	13	1264.50	1288.00
6	R1-07	11	1122.15	1144.76	11	**1108.11**	1119.46
7	R1-08	10	1013.6	1030.05	10	**994.68**	1019.46
8	R1-09	13	1181.01	1213.14	13	**1168.91**	1207.90
9	R1-10	12	1117.01	1148.63	12	**1108.22**	1143.02
10	R1-11	12	1099.26	1133.51	12	**1080.84**	1129.41
11	R1-12	11	1009.68	1039.33	**10**	**992.22**	1031.52
12	R2-01	7	1197.87	1212.23	7	**1197.09**	1223.15
13	R2-02	6	1107.51	1123.12	6	**1092.22**	1132.15
14	R2-03	4	984.85	999.54	5	**983.06**	1003.15
15	R2-04	4	**803.47**	840.90	4	845.30	889.45
16	R2-05	6	**993.42**	1012.96	**5**	999.54	1039.66
17	R2-06	4	959.98	982.33	4	**955.94**	982.89
18	R2-07	3	934.37	943.76	4	**903.59**	948.71
19	R2-08	3	**747.15**	822.57	3	769.96	836.91
20	R2-09	5	944.48	960.25	5	**935.57**	1023.90
21	R2-10	5	994.29	1017.15	**6**	**988.34**	892.93
22	R2-11	4	**837.72**	873.27	**3**	867.95	965.32
23	C1-01	10	828.94	828.94	10	**828.94**	828.94
24	C1-02	10	828.94	845.55	10	**828.94**	835.94
25	C1-03	10	849.86	876.02	10	**835.71**	883.52
26	C1-04	10	886.24	922.50	10	**885.06**	924.82
27	C1-05	10	828.94	831.13	10	**828.94**	829.83
28	C1-06	10	828.94	833.14	10	**828.94**	829.72
29	C1-07	10	828.94	830.09	10	**828.94**	830.09
30	C1-08	10	833.98	853.39	10	**831.73**	845.16
31	C1-09	10	852.98	883.69	10	**840.66**	879.94
32	C2-01	3	**591.56**	591.56	3	**591.56**	591.56
33	C2-02	3	**591.56**	593.16	3	**591.56**	594.43
34	C2-03	3	600.54	616.39	3	**593.21**	613.23
35	C2-04	3	610.01	648.57	3	**606.90**	647.60
36	C2-05	3	**588.88**	596.10	3	**588.88**	601.58
37	C2-06	3	**588.88**	601.49	3	**588.88**	605.03
38	C2-07	3	**589.58**	601.60	3	590.59	599.16
39	C2-08	3	**591.65**	613.47	3	593.15	617.87
40	RC1-01	15	1644.82	1674.45	15	**1637.40**	1671.10
41	RC1-02	14	1495.18	1508.13	14	**1486.85**	1508.40
42	RC1-03	12	1307.59	1356.70	12	**1299.38**	1350.67
43	RC1-04	10	1215.82	1233.24	10	**1200.60**	1228.63
44	RC1-05	15	1539.48	1587.19	15	**1535.80**	1576.40
45	RC1-06	13	**1402.4**	1444.13	13	1403.07	1432.51
46	RC1-07	12	1258.31	1309.30	12	**1230.32**	1285.73
47	RC1-08	11	1171.61	1222.96	11	1165.17	1205.62
48	RC2-01	6	1321.07	1340.22	7	**1315.57**	1350.37
49	RC2-02	5	1185.44	1190.93	5	**1169.72**	1205.30
50	RC2-03	4	1030.94	1030.05	4	**1010.74**	1032.98
51	RC2-04	4	**864.6**	918.12	4	890.28	925.76
52	RC2-05	7	**1211.01**	1254.57	7	1221.28	1266.01
53	RC2-06	6	1112.38	1153.15	6	**1097.65**	1160.59
54	RC2-07	5	1042.65	1063.83	5	**1024.17**	1080.10
55	RC2-08	4	878.87	913.69	4	**864.56**	918.82

To show the difference between the two algorithms, Table 3 represents the best and average distance for the Solomon instances, from which it can be seen that the adaptive BCO-SIH algorithm shows significant differences in most instances. Furthermore, the number of vehicles is also less than or equal.

**Fig 7:**
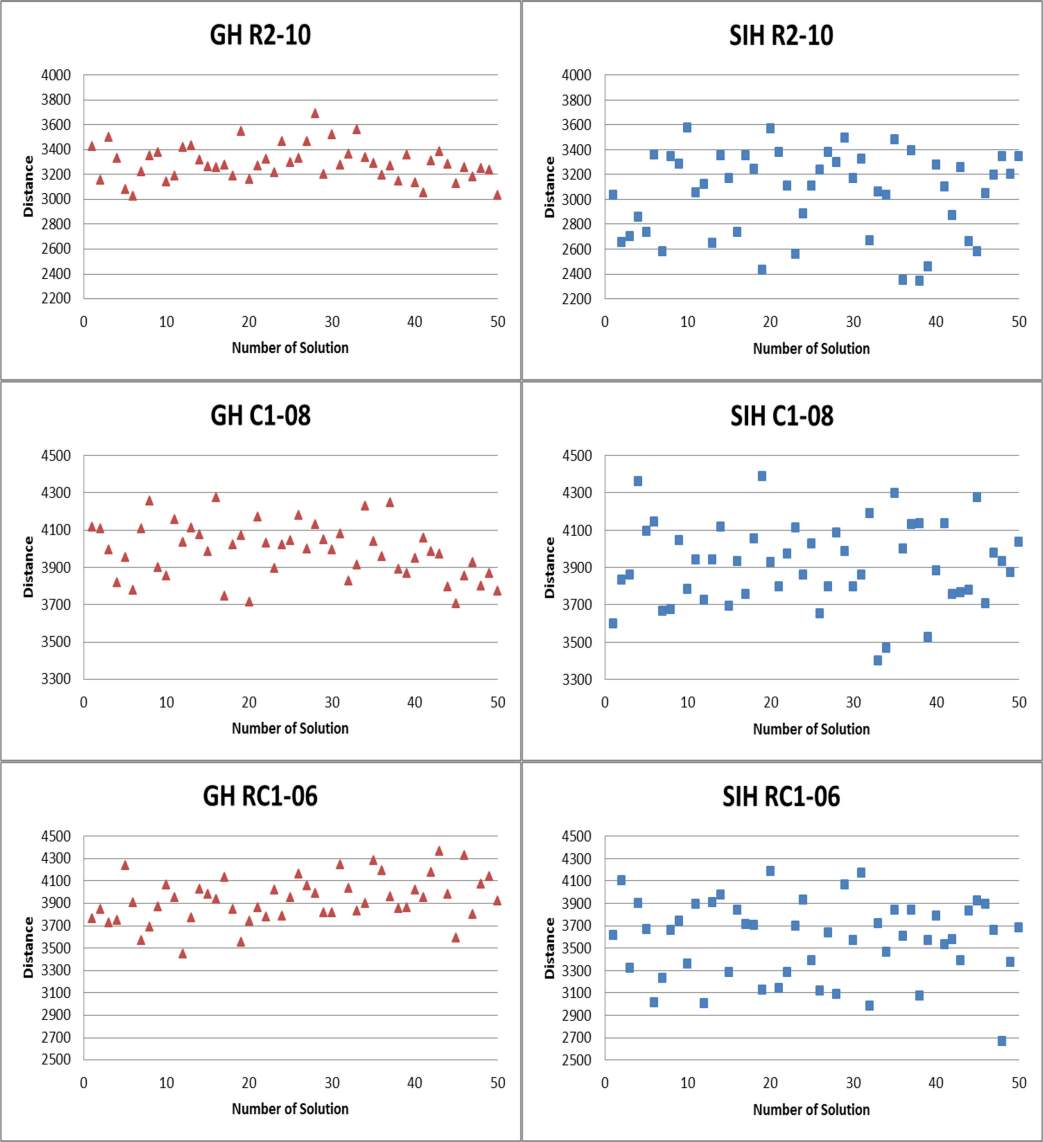
Comparison of distribution of initial population of adaptive BCO-SIH algorithm and that of adaptive BCO-GH algorithm.

Fig 7 represents the distribution of solutions in the initial population of the adaptive BCO-SIH and BCO-GH algorithms for the randomly selected instances, R2-10, C1-08, C2-04, and RC1-06. The figure shows that the diversity of the solutions of the adaptive BCO-SIH algorithm is better than that of the adaptive BCO-GH algorithm. The x-axis represents the number of solutions in the population while the y-axis represents the penalty cost (distance). The quality of solutions in the initial population of the adaptive BCO-GH algorithm is represented by the triangle symbol while that of the adaptive BCO-SIH algorithm is represented by the square symbol.

We also observe that the elements of the generated initial population of the adaptive BCO-SIH algorithm (as represented by the square symbol) for all the datasets are scattered far from one another, representing the distance of the quality of solutions in the population. This phenomenon is perhaps not surprising because the initial solutions are generated by the SIH as long as the hard constraint(s) is (are) respected without considering the violation of the soft constraint(s). For the greedy heuristic initialisation, the distribution of solutions (represented by the triangle symbol) is less scattered than for the SIH, indicating that the method indeed improves the quality of solutions.

### 6.5 Comparison of the Adaptive BCO-SIH and Other State-of-the-Art Approaches

Table 4 compares the results of different heuristics in the literature with those of the adaptive BCO-SIH and BCO-GH based on the average distance in each category. The table shows that the adaptive BCO-SIH algorithm leads with the three new best average results in categories R1, RC1 and RC2. In addition, it is clear that the summation of the average distances for that algorithm is the best.

**Table 4:** Comparison of performance of different approaches against that of adaptive BCO-SIH and adaptive BCO-GH algorithms.

Problem Class	Braysy andGendreu [27]	Schulze andFahle [28]	Potvin andBengio [29]	Ho et al.[30]	Lauet al. [31]	Adaptive BCO-GH	Adaptive BCO-SIH
**C1**	**828.38**	828.94	838.00	833.32	832.13	840.86	837.54
**C2**	589.85	589.93	589.90	593.00	**589.86**	594.08	593.09
**R1**	1222.12	1268.42	1296.83	1203.32	1211.55	1215.51	**1205.91**
**R2**	975.11	1055.90	1117.70	**951.17**	1001.12	955.01	958.05
**CR1**	1389.58	1396.07	1446.20	1382.06	1418.77	1379.40	**1369.82**
**CR2**	1128.38	1308.31	1360.60	1132.79	1170.93	1080.87	**1074.24**
**Sum**	6133.42	6447.57	6649.23	6095.66	6224.36	6065.73	**6038.65**

To show the significant differences between the proposed BCO-SIH algorithm and some approaches in the literature we analyse the results obtained by the proposed algorithm by conducting a statistical test, namely the Friedman’s test. Table 5 summarises the rankings obtained by Friedman’s test.

**Table 5:** Average rankings of compared algorithms and adaptive BCO-SIH (Friedman’s test).

Algorithm	Ranking
**Braysy and Gendreu**	2.42
**Ho et al.**	2.67
**Adaptive BCO-SIH**	2.83
**Lau et al.**	3.42
**Schulze and Fahle**	4.17
**Potvin and Bengio**	5.50

Table 5 shows that Braysy and Gendreu's results rank first, then those of Ho et al., followed by the adaptive BCO-SIH in third place. The results of Lau et al., Schulze and Fahle, Potvin and Bengio’s rank fourth, fifth and sixth, respectively. The p-value computed by the Friedman’s test is 0.0407, which is below the significance interval of 95% (α = 0.05). This value shows that there is a significant difference among the observed results.

We also perform the Holm’s procedure to determine whether there are significant differences between the control algorithm (the best-performing one) and the others. Table 6 shows the significance of the compared algorithms and the control algorithm [27]. The table contains the following columns: i is the ranking for the algorithm, p is the p-value, Holm is the Holm’s procedure value, and in the last column, the null hypothesis is recorded as significant if the p-value ≤ Holm’s value, else it is recorded as not significant. Holm’s procedure rejects those hypotheses that have a p-value ≤ Holm’s procedure value.

**Table 6:** Holm table for α = 0.05 (Friedman’s test).

i	Algorithm	p	Holm	Null hypothesis
1	**Ho et al.**	0.8170	0.0500	Not significant
2	**Adaptive BCO-SIH**	0.6997	0.0250	Not significant
3	**Lau et al.**	0.3545	0.0167	Not significant
4	**Schulze and Fahle**	0.1052	0.0125	Not significant
5	**Potvin and Bengio**	0.0043	0.0100	Significant

The column null hypothesis shows the significant test, only one algorithm shows that there is significant and others show there are no significant, including our algorithm, this means the average results between these algorithms are close to the control algorithm results. However, as shown in column I, our algorithm is second ranked.

Table 7 compares the best-known results of the adaptive BCO-SIH algorithm with those in the literature. The best distance is presented in bold. Distance is used as the main objective (single objective) when comparing the performance of the various algorithms.

**Table 7:** Comparison of best-known results in the literature with those of adaptive BCO-SIH.

		Best-known result			Adaptive BCO-SIH	
No	Instance	N.V	Distance	Source	N.V	Distance
0	R1-01	18	1607.7	Desrochers et al. [32]	20	1643.18
1	R1-02	17	1434.00	Desrochers et al. [32]	18	1476.11
2	R1-03	13	1175.67	Lau et al. [33]	14	1245.86
3	R1-04	10	982.01	Rochat and Tailard [34]	11	1026.91
4	R1-05	15	1346.12	Kallehauge et al. [35]	15	1361.39
5	R1-06	13	1234.6	Cook and Rich [36]	13	1264.50
6	R1-07	11	1051.84	Kallehauge et al. [35]	11	1108.11
7	R1-08	9	960.88	Berger et al. [37]	10	994.68
8	R1-09	12	1013.2	Chiang and Russel [38]	13	1168.91
9	R1-10	12	1068	Cook and Rich [36]	12	1108.22
10	R1-11	12	1048.7	Cook and Rich [36]	12	1080.84
11	R1-12	10	953.63	Rochat and Tailard [34]	10	992.22
12	R2-01	4	1252.37	Homberger and Gehring [39]	7	**1197.09**
13	R2-02	3	1158.98	Lau et al. [33]	6	**1092.22**
14	R2-03	3	939.5	Woch and Lebkowski [40]	5	983.06
15	R2-04	2	825.52	Bent and Van [41]	4	845.30
16	R2-05	3	994.42	Rousseau et al. [42]	5	999.54
17	R2-06	3	833.00	Thangiah et al. [43]	4	955.94
18	R2-07	3	814.78	Rochat and Tailard [34]	4	903.59
19	R2-08	2	726.75	Mester et al. [44]	3	769.96
20	R2-09	3	855	Thangiah et al. [43]	5	935.57
21	R2-10	3	939.34	Mester et al. [44]	6	988.34
22	R2-11	2	885.71	Woch and Lebkowski [40]	3	**867.95**
23	C1-01	10	827.30	Desrochers et al. [32]	10	828.94
24	C1-02	10	827.30	Desrochers et al. [32]	10	828.94
25	C1-03	10	826.30	Tavares et al. [45]	10	835.71
26	C1-04	10	822.90	Tavares et al. [45]	10	885.06
27	C1-05	10	827.30	Tavares et al. [45]	10	828.94
28	C1-06	10	827.30	Desrochers et al. [32]	10	828.94
29	C1-07	10	827.30	Tavares et al. [45]	10	828.94
30	C1-08	10	827.30	Tavares et al. [45]	10	831.73
31	C1-09	10	827.30	Tavares et al. [45]	10	840.66
32	C2-01	3	589.10	Cook and Rich [36]	3	591.56
33	C2-02	3	589.10	Cook and Rich [36]	3	591.56
34	C2-03	3	591.17	Li and Lim [46]	3	593.21
35	C2-04	3	590.60	Potvin and Bengio [29]	3	606.90
36	C2-05	3	**588.88**	De Backer et al. [47]	3	**588.88**
37	C2-06	3	588.49	Lau et al. [33]	3	588.88
38	C2-07	3	588.29	Rochat and Tailard [34]	3	590.59
39	C2-08	3	588.32	Rochat and Tailard [34]	3	593.15
40	RC1-01	15	1619.8	Kohl et al. [48]	15	1637.40
41	RC1-02	13	1530.86	Cordone and Wolfler [49]	14	**1486.85**
42	RC1-03	11	1261.67	Shaw [50]	12	1299.38
43	RC1-04	10	1135.48	Cordeau et al. [51]	10	1200.60
44	RC1-05	13	1629.44	Berger et al. [37]	15	**1535.80**
45	RC1-06	12	1395.4	Chiang and Russel [38]	13	1403.07
46	RC1-07	11	1230.5	Taillard et al. [52]	12	**1230.32**
47	RC1-08	10	1139.8	Taillard et al. [52]	11	1165.17
48	RC2-01	4	1249	Thangiah et al. [43]	7	1315.57
49	RC2-02	4	1164.3	Taillard et al. [52]	5	1169.72
50	RC2-03	3	1049.62	Czech and Czarnas [53]	4	**1010.74**
51	RC2-04	3	798.41	Mester et al. [44]	4	890.28
52	RC2-05	4	1297.19	Mester et al. [44]	7	**1221.28**
53	RC2-06	3	1146.32	Homberger [54]	6	**1097.65**
54	RC2-07	3	1061.14	Zbigniew et al. [55]	5	**1024.17**
55	RC2-08	3	828.14	Ibaraki et al. [56]	4	864.56

The adaptive BCO-SIH algorithm achieves low distance routing results for 11 out of 56 datasets. This indicates that 20% obtained a lower distance than in the best-known solutions gained from various heuristics over the years. Furthermore, the results of our proposed algorithm for total distance minimisation of VRPTW are better or equal to those of the best separately available results. The adaptive BCO-SIH algorithm also yields more competitive route solutions for 25 instances with a similar number of vehicles than the best-known VRPTW solutions in the literature.

## Conclusion

The adaptive BCO algorithm proposed in this study for the VRPTW problem excels in terms of both the improved quality of its solutions and shorter computational time. The results therefore show that the proposed algorithm is effective and has considerable potential for solving the VRPTW problem. The reasons for this are twofold: the use of an initialisation strategy (SIH) improves the diversification of the initial population and the online parameter tuning strategy chooses suitable parameter values based on the search progress.

A comparison of the results of the proposed algorithm with those of some state-of-the-art approaches reveals that the adaptive BCO-SIH algorithm can obtain 11 new best results in Solomon’s 56 VRPTW 100 customer instances, which corresponds to 20% and three out of six categories. This methodology is therefore highly suitable for the VRPTW problem. In our future work, we aim to investigate the performance of this approach and attain a balance between exploration and exploitation by enhancing the BCO-SIH algorithm through hybridisation with local search algorithms.
